# Exploring Relationships Between Anthropometry, Body Composition, Maturation, and Selection for Competition: A Study in Youth Soccer Players

**DOI:** 10.3389/fphys.2021.651735

**Published:** 2021-03-11

**Authors:** Filipe Manuel Clemente, Cain C. T. Clark, César Leão, Ana Filipa Silva, Ricardo Lima, Hugo Sarmento, António J. Figueiredo, Thomas Rosemann, Beat Knechtle

**Affiliations:** ^1^Escola Superior Desporto e Lazer, Instituto Politécnico de Viana do Castelo, Rua Escola Industrial e Comercial de Nun’Álvares, Viana do Castelo, Portugal; ^2^Instituto de Telecomunicações, Delegação da Covilhã, Lisbon, Portugal; ^3^Centre for Intelligent Healthcare, Coventry University, Coventry, United Kingdom; ^4^The Research Centre in Sports Sciences, Health Sciences and Human Development, Vila Real, Portugal; ^5^N2i, Polytechnic Institute of Maia, Maia, Portugal; ^6^Research Unit for Sport and Physical Activity, Faculty of Sport Sciences and Physical Education, University of Coimbra, Coimbra, Portugal; ^7^Institute of Primary Care, University of Zurich, Zurich, Switzerland; ^8^Medbase St. Gallen Am Vadianplatz, St. Gallen, Switzerland

**Keywords:** football, young, maturation, performance, talent development, motor development

## Abstract

**Purpose:**

The purpose of this study was to analyze variations of selection for competition between late and early mature players and test the relationships between anthropometry, body composition, maturation, and selection for competition.

**Methods:**

Seventy-nine youth soccer players from under-11 to under-14 participated in this study, over 6 months. Body composition and maturity offset were estimated based on anthropometric data collected. Participants were also monitored for their number of matches as starters and time of play accrued in minutes.

**Results:**

Minutes played had large correlation coefficients with maturity offset (*r* = 0.58), and leg length and sitting height interaction (*r* = 0.56). Multiple linear regression explained 35% of the variation in minutes played (*p* < 0.001, *R*^2^ = 0.41, *R*^2^_adjusted_ = 0.35, RMSE = 334.8), but only 12% of the variation in matches as starter (*p* = 0.04, *R*^2^ = 0.21, *R*^2^_adjusted_ = 0.12, RMSE = 5.47) between above and below the median of the maturity offset was accounted for, respectively.

**Conclusions:**

Although maturation may play a role in the minutes of play accrued and matches as starters in young, it is not necessarily determining. A significant amount of the variation in the minutes of play accrued of players can be accounted for when considering body composition and anthropometric data.

## Introduction

Children at the same chronological age may vary meaningfully in maturation status, where some children can be more or less advanced in terms of maturation in comparison with their peers (Baxter-Jones et al., 2005). In the case of youth team sports (e.g., soccer), this evidence is extremely important, mainly because in critical periods (between 10 and 15 years old) the inter-player variation of maturation status can be very high (Figueiredo et al., 2009b, 2020). Such variation should be considered by coaches, among others, to standardize the training process to the biological maturation status (Lloyd et al., 2014). Moreover, talent identification and selection should also consider maturation status as a critical parameter to make reasonable decisions (Gonçalves et al., 2012). These considerations have a higher extent because it is about 14–15 years old that selection/rejection for the soccer system mainly occurs (Malina et al., 2015). For those reasons, assessment of maturation in youth athletes is regarded as a determinant, and has been conducted using different approaches such as, among others, skeletal age, pubertal status, estimated age at peak height velocity (PHV), age at menarche, or determination of maturity status using the secondary sex characteristics (Malina et al., 2012, 2015).

The impact of maturation on multiple dimensions of players’ performance has been extensively researched (Vandendriessche et al., 2012; Gastin et al., 2013). For example, a study conducted in 78 under-15 and under-16 male soccer players (Vandendriessche et al., 2012) revealed that more mature players had greater morphological measures and outperformed their later maturing teammates in fitness parameters (e.g., handgrip strength, standing broad jump, countermovement jump, agility T-test or sprint). Moreover, earlier maturers also seem to have an advantage in running fitness and performance (Gastin et al., 2013). Despite these apparent advantages on fitness parameters, in other indicators, the influence of maturation is less visible. In a study testing the balance of youth players, later maturers appear to have an advantage in static and dynamic postural control due to the adolescent awkwardness that may cause temporary balance deficits (John et al., 2019). Despite these findings, opposing evidence has been reported in youth players, suggesting that stabilometric parameters improved with age until zero maturity offset (Zago et al., 2020). In other dimensions of performance, such as motor coordination, no meaningful impact appears to be conferred due to maturation (Vandendriessche et al., 2012). Moreover, a study conducted in 48 under-15 male soccer players also revealed a limited influence of maturity, growth status, or functional fitness on the tactical performance and tactical knowledge of players (Borges et al., 2018).

Comparisons between players, considering the maturation status, have not been limited to the fitness, coordination, and technical/tactical levels. Indeed, some studies have sought to analyze the possible effects of maturation on soccer match performance (Goto et al., 2019; Lovell et al., 2019). Comparisons of match performance between earlier and later maturers at different age groups (Goto et al., 2019) revealed that earlier maturers covered significantly greater distances than later maturers in under-9/under-10 competitive levels. Moreover, earlier maturers also covered greater distances, particularly at high-speed running, in the under-13/under-14 groups (Goto et al., 2019). Contradictorily, a study conducted in under-15 players (Lovell et al., 2019) revealed that later maturing players accrued substantially more higher intensity running.

Considering the possible influence of maturation on the selection by coaches to use more or less mature players in matches, a study conducted in different age groups revealed that only in under-9 and -10 was found significant evidence that earlier maturers had longer pitch time than later maturers (Goto et al., 2019). In the remaining competitive levels, under-11 to under-14, there were no meaningful differences in playing time (Goto et al., 2019). The option to use more or less mature players in matches during a critical period of development may lead to player promotion and/or dropout. Indeed, studies on soccer have suggested a consistent exclusion of later mature players as age- and sport-specific demands increase, mainly in the critical period of adolescence (Figueiredo et al., 2009a). Moreover, some studies have suggested that athletes have been chosen largely according to their maturation status (Johnson et al., 2017).

Selection of players based on temporary physical advantage may lead to considerable heterogeneities in the number of matches and playing time accrued during adolescence. Despite that, few studies have been analyzing the influence of maturation on the number of matches performed or accumulated playing time (Gravina et al., 2008; Coelho e Silva et al., 2010; Goto et al., 2019). Moreover, interactional factors, height, body mass, or body composition may also interfere in the criteria to select players for the team lineup and in accruing more or less match time. Thus, it is important to determine the interactional effect of maturation status, anthropometry, and body composition on accumulated playing time and the number of matches played as a starter in youth teams. Such information would help to facilitate understanding of the aforementioned factors and players’ selection practices. Therefore, the purpose of this study was (1) to analyze variations of time of play accrued (within-age category) between players above and below the median of maturity offset in young soccer players from under-11 to under-14 levels; (2) to analyze the relationships between anthropometrics, body composition, maturity offset, and minutes/participation in match; and (3) to determine the influence of anthropometry, body composition, and maturation (maturity offset) on minutes/participation in match.

## Materials and Methods

### Participants

Seventy-nine youth male Portuguese soccer players (under-11: *N* = 16, age: 10.4 ± 0.3 years; under-12: *N* = 20, age: 11.6 ± 0.3 years; under-13: *N* = 22, age: 12.4 ± 0.4 years; and under-14: *N* = 21, age: 13.5 ± 0.3 years) participated in this study. Players were a member of each team during the entire period of analysis. The inclusion criteria used were (1) players who had no injuries during the period of data collection and (2) players who participated in 80% of the training sessions or above during the period of data collection. As exclusion criteria, the option was (1) players who gave up or moved to another team during the data collection, and (2) players who interrupted participation in training sessions for more than 1 week. Players and their parents/legal guardians were informed about the study design and methodological approach in a meeting made with researchers and staff at the beginning of the season. After agreement of players and their parents/legal guardians, an informed consent was signed by the parents/legal guardians. The study was approved by the local university scientific council and followed the ethical standards of the declaration of Helsinki for the study in humans.

### Experimental Approach

The study followed a cohort design and occurred between early September 2019 and end of February 2020. During the period of observation, the number of matches as starters (part of the starting lineup) and time played (sum of all minutes played during the period) were registered for each player. In the middle of the data collection period (December 9–12, 2019), the assessment of anthropometric variables [height, sitting height, leg length, body mass, waist circumference (WC), and four skinfolds] was carried out. Aiming to ensure similar conditions between teams, a period of 48 h between the last match and the assessment was observed. Moreover, the assessment was conducted at the same period of the day (afternoon, ∼17:00) in a controlled environment (room, with a stable temperature of 23°C and relative humidity of 55%).

### Anthropometry

The measurements were conducted in an appropriate room before the participants’ first training session. All subjects wore light clothing and were barefooted, following which their height and sitting height were measured to the nearest 0.1 cm with a portable stadiometer (Seca 206, Hamburg, Germany); sitting height was subtracted from height to estimate leg length (0.1 cm); and body mass was assessed to the nearest 0.1 kg with a Prozis Smart Scale—Sensit. In addition, waist circumference measurement was taken with the participant in a standing position, over the naked skin, to the nearest 0.1 cm using a Cescorf anthropometric tape measure; the tape was applied horizontally, just above the umbilicus, as this has been demonstrated to be a good marker of obesity risk (Andaki et al., 2018). The mean of two measurements was considered for all the variables.

### Body Composition

Four skinfolds (triceps, suprailiac, supraspinal, and calf) were assessed twice (at nearest 1 mm) with a Harpenden caliper (British Indicators, London, United Kingdom), and the mean of the two measurements was considered to estimate body fat. Despite only two were used for the calculation of body fat (BF), the four were extracted based on different possibilities for calculation. The Lozano-Berges equation (Lozano-Berges et al., 2018) was used to estimate BF. The equation combines the sex, triceps, and iliac-crest skinfold thickness (Lozano-Berges et al., 2018).

The procedure of measuring the skinfolds was performed by a certified tester, following the International Society for the Advancement of Kinanthropometry recommendations (Stewart et al., 2011). An intra-observer reliability analysis was conducted using two assessments for each skinfold in a sample of 10 players. The intra-class correlation (ICC) test was executed to test the reliability in the two assessments. The results revealed a mean (among the four skinfolds) ICC level of 0.94, suggesting an excellent reliability level on data collection of the skinfolds.

### Maturity Offset

The length of time (age in years until the moment of assessment), weight (body mass), height, sitting height, and leg length were included in the equation to determine the age at the PHV (Mirwald et al., 2002): −(9.236 + 0.0002708 × leg length and sitting height interaction) − (0.001663 × age and leg length interaction) + (0.007216 × age and sitting height interaction) + (0.02292 × weight by height ratio). This specific equation was tested in the original article (Mirwald et al., 2002) and revealed an SE of 0.57 years in males. Per each team (competitive levels), the median of maturity offset was determined and defined two groups: (1) the players above the median of maturity offset (defined as late mature) and (2) the players below the median (defined as early mature).

### Participation in Matches

Under-11 and under-12 teams participated in a regional competition in which the seven vs. seven is the official format of play, the time of play lasts 60 min (two halves of 30 min), and the coach may perform as many replacements as they want (including a player who may re-enter after being replaced). The under-13 and under-14 teams participated in regional and national competition, respectively, in which the format of play is the eleven vs. eleven and the time of play lasts 70 min (two halves of 35 min). In under-13 and under-14 teams, it is only possible to do five replacements and a player cannot re-enter when replaced.

The number of occasions in which a player started the match in the starting lineup was registered by the researchers. Moreover, the match time (min: minutes) of the player was also monitored and registered. The sum of matches as starter (n) and time played (min) was registered per player.

### Statistical Procedures

Descriptive statistics were computed to ascertain the mean ± SD for all variables. Normality and homogeneity of the sample were preliminarily tested and confirmed in the Kolmogorov–Smirnov and Levene’s tests, respectively (*p* > 0.05). Subsequently, one-way multiple analysis of variance (MANOVA) was conducted to investigate the interactions between competitive level and group below/above maturity offset median for the variables of number of matches as starter and minutes played, respectively. Comparisons of measures between players above or below maturity offset median were conducted using an independent *t*-test, followed by the calculation of standardized effect size of Cohen (*d*). The magnitude of differences was interpreted based on the following thresholds (Batterham and Hopkins, 2006): trivial (*d* ≥ 0.0 but <0.2), small (*d* ≥ 0.2 but <0.6); moderate (*d* ≥ 0.6 but <1.2); large (*d* ≥ 1.2).

Next, pairwise correlation, using Pearson’s correlation coefficients, was conducted, with relationships reported as *R* with associated *p* value, and described using nomenclature recommended by Cohen et al. (2003), i.e., small (*R* ≥ 0.1 but <0.3), medium (*R* ≥ 0.3 but <0.5), and large (*R* ≥ 0.5). Multiple linear regression, including competitive level, BMI, maturity offset, lean mass, and leg length and sitting height interaction as covariates, was conducted to explain the variance between below and above the median of the maturity offset. In all instances, statistical significance was accepted, *a priori*, at *p* < 0.05, and all analyses were conducted in R (R Core Team, 2018), using the *car: companion to applied regression package* (Fox and Weisberg, 2018) and jamovi software extension (The Jamovi Project, 2019).

## Results

Basic descriptive statistics of anthropometry, body composition, maturation, and matches of players are detailed in Table 1.

**TABLE 1:** Descriptive statistics (mean ± SD) of anthropometrics, body composition, maturation, and matches of players.

	Under-11	Under-12	Under-13	Under-14
*N*	16	20	22	21
Age (years)	10.4 ± 0.3	11.6 ± 0.3	12.4 ± 0.4	13.5 ± 0.3
Height (cm)	139.0 ± 5.3	142.9 ± 5.4	149.6 ± 8.1	162.1 ± 5.8
Sitting height (cm)	73.2 ± 5.7	73.5 ± 3.4	77.8 ± 3.6	83.0 ± 3.7
Leg length (cm)	65.8 ± 6.7	69.4 ± 3.1	71.8 ± 4.8	79.0 ± 3.6
Body mass (kg)	35.7 ± 5.8	387 ± 5.5	41.9 ± 7.1	52.5 ± 6.9
BMI (kg/m^2^)	18.4 ± 2.6	18.9 ± 1.6	18.7 ± 2.2	19.9 ± 1.8
Waist circumference (cm)	62.0 ± 4.8	63.1 ± 3.9	65.2 ± 5.0	67.9 ± 3.9
Fat mass (%)	18.4 ± 3.8	18.8 ± 3.9	18.4 ± 4.0	17.1 ± 2.1
Lean mass (kg)	21.8 ± 1.7	22.8 ± 1.7	24.8 ± 2.1	27.4 ± 2.0
BMHR (kg/cm)	25.6 ± 3.7	27.04 ± 3.03	27.9 ± 3.8	32.3 ± 3.5
LLSH (A.U.)	4794.8 ± 412.3	5106.6 ± 380.7	5597.7 ± 612.7	6565.0 ± 458.4
Maturity offset (years)	−3.0 ± 0.5	−2.4 ± 0.4	−1.6 ± 0.5	−0.4 ± 0.5
Age of PHV (years)	13.4 ± 0.6	14.0 ± 0.4	14.0 ± 0.4	13.9 ± 67.9
MS (n)	7.0 ± 4.9	6.6 ± 4.1	11.9 ± 5.4	12.05 ± 7.5
MP (min)	420.0 ± 175.3	400.8 ± 185.1	694.0 ± 270.2	962.6 ± 533.5

*N, number; MS, match as starter; MP, minutes played; BMI, body mass index; LLSH, leg length and sitting height interaction; PHV, peak height velocity; BMHR, body mass/height ratio; A.U., arbitrary units.*

One-way MANOVA revealed non-significant interactions between competitive level and group below/above maturity offset median for the variables of number of matches as starter (*p* = 0.151; SE = 0.071) and minutes played (*p* = 0.125; SE = 0.077). Comparisons of number of matches as starter and minutes played between players below and above the median of maturity offset can be found in Table 2.

**TABLE 2:** Comparisons of number of matches as starter (MS) and minutes played (MP) between players below (early maturers) and above (late maturers) the median of maturity offset.

	Median of maturity offset		Below median	Above median	% of difference (above − below)	*p*	d	Magnitude
Under-11	−3.0	MS (n)	6.6 ± 4.8	7.4 ± 5.3	4.0 ± 113.4	0.771	0.05 ± 1.21	*Trivial*
		MP (min)	427.5 ± 175.8	412.5 ± 186.6	−6.6 ± 67.8	0.871	−0.10 ± 1.03	*Trivial*
Under-12	−2.3	MS (n)	6.0 ± 3.7	7.3 ± 4.7	14.6 ± 80.4	0.482	0.22 ± 1.04	*Small*
		MP (min)	390.5 ± 186.2	413.3 ± 194.1	3.5 ± 46.8	0.792	0.08 ± 1.00	*Trivial*
Under-13	−1.6	MS (n)	12.9 ± 4.9	10.6 ± 5.9	−30.5 ± 48.6	0.327	−0.75 ± 1.34	*Moderate*
		MP (min)	755.9 ± 234.4	619.7 ± 303.3	−26.7 ± 39.8	0.248	−0.80 ± 1.34	*Moderate*
Under-14	−0.4	MS (n)	14.4 ± 7.8	8.3 ± 5.7	−4.4 ± 60.5	0.069	−0.05 ± 0.61	*Trivial*
		MP (min)	1126.5 ± 558.2	696.1 ± 384.6	−44.8 ± 64.1	0.052	−0.66 ± 1.10	*Moderate*

Despite no significant differences, late mature under-13 players played fewer as starters (−30.5%; *p* = 0.327; *d* = −0.75, moderate effect) and less minutes (−26.7%; *p* = 0.248; *d* = −0.80, moderate effect) than early mature teammates. In under-14, late mature players played less time than early mature ones (−44.8%; *p* = 0.052; *d* = −0.66, moderate effect).

Pairwise correlations of all variables are presented in Table 3, presented as Pearson’s R with associated p value. Minutes played had large correlation coefficients with maturity offset (*r* = 0.58, 95% CI [0.41, 0.71]) and leg length × sitting height interaction (*r* = 0.56; 95% CI [0.39, 0.70]).

**TABLE 3:** Correlation coefficients (*r*) between anthropometric variables, maturity offset, and minutes played/matches as starter.

		Age (years)	Height (cm)	Sitting height (cm)	Leg length (cm)	Body mass (kg)	BMI (kg/m^2^)	WC (cm)	Fat mass (%)	BMHR (kg/cm)	LLSH	Maturity offset	Age at PHV (years)	MS (n)
Height (cm)	Pearson’s *r*	**0.808**	–											
	*p* value	<0.0001***	–											
Sitting height (cm)	Pearson’s *r*	**0.6709**	**0.8507**	–										
	*p* value	<0.0001***	<0.0001***	–										
Leg length (cm)	Pearson’s *r*	**0.7351**	**0.8922**	**0.5217**	–									
	*p* value	<0.0001***	<0.0001***	<0.0001***	–									
Body mass (kg)	Pearson’s *r*	**0.7029**	**0.8661**	**0.7536**	**0.7583**	–								
	*p* value	<0.0001***	<0.0001***	<0.0001***	<0.0001***	–								
BMI (kg/m^2^)	Pearson’s *r*	0.2676	0.3225	0.2983	0.2672	**0.7448**	–							
	*p* value	0.0171*	0.0037**	0.0076**	0.0173*	<0.0001***	–							
WC (cm)	Pearson’s *r*	0.466	**0.5604**	0.4653	**0.5099**	**0.8385**	**0.8663**	–						
	*p* value	<0.0001***	<0.0001***	<0.0001***	<0.0001***	<0.0001***	<0.0001***	–						
Fat mass (%)	Pearson’s *r*	−0.1035	0.0001	0.0634	−0.0542	0.342	**0.6977**	**0.5656**	–					
	*p* value	0.3643	0.999	0.5788	0.635	0.002**	<0.0001***	<0.0001***	–					
BMHR (kg/cm)	Pearson’s *r*	**0.5875**	**0.7209**	**0.6333**	**0.626**	**0.9688**	**0.8866**	**0.9012**	**0.5006**	–				
	*p* value	<0.0001***	<0.0001***	<0.0001***	<0.0001***	<0.0001***	<0.0001***	<0.0001***	<0.0001***	–				
LLSH (A.U.)	Pearson’s *r*	**0.8101**	**0.9967**	**0.8151**	**0.9175**	**0.8641**	0.3201	**0.5601**	−0.0186	**0.7181**	–			
	*p* value	<0.0001***	<0.0001***	<0.0001***	<0.0001***	<0.0001***	0.004**	<0.0001***	0.8705	<0.0001***	–			
Maturity offset (years)	Pearson’s *r*	**0.8997**	**0.9258**	**0.9198**	**0.7124**	**0.8424**	0.3693	**0.5587**	0.0157	**0.7211**	**0.9104**	–		
	*p* value	<0.0001***	<0.0001***	<0.0001***	<0.0001***	<0.0001***	0.0008***	<0.0001***	0.8905	<0.0001***	<0.0001***	–		
Age at PHV (years)	Pearson’s *r*	0.3168	−0.1762	−0.4713	0.1188	−0.2457	−0.2164	−0.1716	−0.2955	−0.2488	−0.1381	−0.1265	–	
	*p* value	0.0045**	0.1203	<0.0001***	0.2969	0.0291*	0.0554	0.1304	0.0082**	0.027**	0.2247	0.2665	–	
MS (n)	Pearson’s *r*	0.3499	0.3465	0.3018	0.3031	0.3103	0.131	0.2386	−0.0674	0.2614	0.3583	0.374	0.0096	–
	*p* value	0.0016**	0.0018**	0.0069**	0.0066**	0.0054**	0.2497	0.0342*	0.555	0.02*	0.0012**	0.0007***	0.9332	–
MP (min)	Pearson’s *r*	**0.5234**	**0.5472**	0.4999	0.4587	0.4935	0.196	0.3285	−0.1066	0.4109	**0.556**	**0.58**	−0.0471	**0.9145**
	*p* value	<0.0001***	<0.0001***	<0.0001***	<0.0001***	<0.0001***	0.0835	0.0031**	0.3499	0.0002***	<0.0001***	<0.0001***	0.6805	<0.0001***

Strong R value denoted as bold; *p < 0.05; **p < 0.01; ***p < 0.001; MS, match as starter; MP, minutes played; BMI, body mass index; WC, waist circumference; LLSH, leg length and sitting height interaction; PHV, peak height velocity; BMHR, body mass/height ratio; A.U., arbitrary units.

Following multiple linear regression, including competitive level, BMI, maturity offset, lean mass, and leg length and sitting height interaction as covariates, 35% of the variation in minutes played [*F*_(6, 50)_ = 5.84, *p* < 0.001, *R*^2^ = 0.41, *R*^2^_adjusted_ = 0.35, RMSE = 334.8] but only 12% of the variation in matches as starter [*F*_(6, 50)_ = 2.31, *p* = 0.04, *R*^2^ = 0.21, *R*^2^_adjusted_ = 0.12, RMSE = 5.47] between above and below median of the maturity offset was accounted for, respectively. For both models, lean mass and leg length × sitting height (LLSH) interaction were the largest contributors to the minutes played (standardized β [95% CI], lean mass = 1.3 [−1.27, 3.88]; LLSH = −1.29 [−4.69, 2.1]) and matches as starter (standardized β [95% CI], lean mass = 2.1 [−0.78, 5.16]; LLSH = −2.57 [−6.49, 1.35]) models, respectively.

## Discussion

The current study sought to determine possible influences of anthropometry, body composition, and maturation on accumulated time of play and matches as starter in young soccer players. The main findings highlighted no significant differences between early or late mature players within each competitive level in the minutes of play and number of matches as starter, despite the result for minutes of play for under-14 that was in the edge of statistical significance. Correlations between measures revealed that minutes of play were strongly correlated with height, leg length and sitting height interaction, and maturity offset. Finally, 35% of the minutes of play were explained by a model including competitive level, BMI, maturity offset, lean mass, and leg length and sitting height interaction as covariates. Moreover, lean mass and leg length and sitting height interaction were the largest contributors in the model.

Biological age is highly variable when closer to the PHV, and for that reason it is extremely important to track maturity offset of young players and control possible benefits of being an early maturer on player’s selection made by coaches (Vandendriessche et al., 2012). It has been well documented, for example, that playing positions in youth soccer are precipitately chosen by morphology that is, directly or indirectly, influenced by maturation (Gravina et al., 2008; Valente-dos-Santos et al., 2012; Towlson et al., 2017). Another example is the relative impact of being a late or early maturer for talent selection and dropout (Vaeyens et al., 2006; Figueiredo et al., 2009a). For example, selected players had an advanced maturity status on under-14 players (Coelho e Silva et al., 2010). Indeed, considering the influence of maturation in the aforementioned examples, the present study had the principal aim of analyzing possible differences of playing time and matches as starter between maturation levels. Aiming to compare early and late maturers within a team, a median value of maturity offset was set and the groups in the team were organized dichotomously. Results revealed no significant differences between groups (i.e., above or below median of maturity offset) and no interaction with the competitive levels analyzed (under-11 to under-14). Despite that, with increasing age, moderate effect sizes revealed a favorable tendency to increase the playing time in the early maturing players. Further, late maturing under-13 and under-14 played proportionally less minutes than the early mature teammates (−26.7 and −44.8%, respectively). Although moderate effect sizes were evident, statistical significance was not found, thus, our findings are in line with a previous study that compared the playing time of earlier and later maturers and revealed that, between under-11 and under-14, no meaningful differences in playing time were verified (Goto et al., 2019). This may suggest that in these particular teams, the choice made by coaches may not be exclusively related with the benefits of being early maturing for the performance in that precise moment. However, this should be interpreted with caution because the guidelines for the specific academy may play an important role in these results. The absence of significant differences in playing time accrued and matches as starter between early and late mature players are in line with the suggestions of previous studies that recommend to make decisions based not exclusively in physical and instantaneous parameters of performance, but also using other selectors, such as skills, motor coordination, or tactics (Larkin and O’Connor, 2017; Sarmento et al., 2018). However, and as mentioned before, of interest is that the age trend (despite the lack of statistical differences) in the number of matches as starter and playing time favored the early maturers, i.e., when the game progresses to a formal format (eleven vs. eleven) and the replacements are no longer reversible, coaches tend to select early maturing more often.

A second objective of the present study was to analyze the relationships between playing time accrued, matches as starter, and the remaining anthropometric and maturation-related variables. Some studies have suggested relationships between anthropometry/body composition and maturation-related variables with technical performance (Moreira et al., 2017), physical performance (Philippaerts et al., 2006), and match-running performance (Lovell et al., 2019). Indeed, considering the influence of anthropometry/body composition and maturity on the aforementioned measures, we hypothesized possible relationships with playing time accrued and matches as starters. The major findings revealed that minutes played had large correlation coefficients with maturity offset, and leg length and sitting height interaction, respectively, thus suggesting that being more mature and having a greater length and sitting height interaction are highly correlated with selection made by coaches to give more minutes in players at young ages. Moreover, height also had a large correlation coefficient with minutes played. To the authors’ knowledge, our study was the first to explore such relationship. The results seem to suggest that being taller and more mature may benefit players to play more, despite that other variables were not assessed, namely, fitness status and technical/tactical skills.

In a study using multiple linear regression with anthropometric, motor coordination, and fitness variables to predict future playing minutes in young soccer players (Deprez et al., 2015), it was revealed that standing broad jump was the most determining measure. In our study, multiple linear regression was used to explore the influence of anthropometry, body composition, and maturity offset on playing time and matches as starter, with results explaining 35% of variation in playing time accrued, but only 12% of matches as starter. In both cases, lean mass and leg length/sitting height interaction were the significant and largest contributors in the models. Interestingly, a study comparing selected and non-selected youth soccer players revealed that fat percentage of selected players was lower than non-selected, thus more lean mass can be considered a determinant for specific cutoffs situations (Lago-Peñas et al., 2014). In fact, it seems that skinfolds may play a negative determining effect on the physical performance of young soccer players (Gil et al., 2014). Moreover, greater lean mass may also contribute to a greater availability to produce greater contractions and have better performance in power-related actions (e.g., jumping, sprinting) (Amonette et al., 2014). Therefore, body composition and anthropometry seem to play a determining role in playing time in youth soccer.

Despite the novelty of the present study, there are some limitations that must be considered. One of the limitations is related to the small sample included. Only one team was analyzed for each competitive level, thus possible inferences should be considered with caution because variables could be influenced by contextual factors. Another limitation is that fitness and technical/tactical variables were not included in the analysis. Finally, this study represented only 6 months of data collection and this should be also considered when making inferences about the data. For those reasons, future studies should consider increasing the size of the sample, as well as the length of data collection, and to add a multidimensional approach, incorporating fitness variables and technical/tactical measures.

Despite limitations, this study is one of the few to provide evidence regarding possible relationships between maturation and anthropometry and the minutes of play accrued and matches as a starter at different competitive levels in youth soccer system. This study will help to facilitate our understanding of player match selection by coaches. Despite the finding that maturation had no strong determinacy in explaining the minutes of play accrued, anthropometry played an important role, and as such, should be considered in practical scenarios. It is advisable that player selections based on momentary conditions should be avoided, and multidimensional aspects related to skill level and, mainly, potential should also be criteria to providing game time and opportunities to young soccer players.

## Conclusion

In conclusion, no significant differences in minutes of play accrued and matches as a starter were found between early and late mature players within under-11 to under-14 competitive levels. In addition, large correlations were found between minutes of play accrued and height, leg length and sitting height interaction, and maturity offset, respectively, in the overall population analyzed. Finally, models to explain the variation of minutes of play and matches as starter accounted for 35 and 12%, respectively, in which the largest contributors to the model were lean mass and leg length and sitting height interaction. Considering the main findings, it is reasonable to assume that maturation is not necessarily a determining factor in the participation of matches, and that anthropometry and body composition may play an important role. However, these conclusions should be interpreted with caution due to the context of this study design.

## Data Availability Statement

The raw data supporting the conclusions of this article will be made available by the authors, without undue reservation.

## Ethics Statement

The studies involving human participants were reviewed and approved by the local University scientific council and followed the ethical standards of Declaration of Helsinki for the study in humans. Written informed consent to participate in this study was provided by the participants’ legal guardian/next of kin.

## Author Contributions

FC conceived and led the project and wrote and revised the original manuscript. CC performed the data analysis and statistical report. CL collected the data wrote and revised the original manuscript. AS, RL, HS, AF, TR, and BK wrote and revised the original manuscript. All authors contributed to the article and approved the submitted version.

## Conflict of Interest

The authors declare that the research was conducted in the absence of any commercial or financial relationships that could be construed as a potential conflict of interest.
